# Rare Genomic Structural Variants in Complex Disease: Lessons from the Replication of Associations with Obesity

**DOI:** 10.1371/journal.pone.0058048

**Published:** 2013-03-12

**Authors:** Robin G. Walters, Lachlan J. M. Coin, Aimo Ruokonen, Adam J. de Smith, Julia S. El-Sayed Moustafa, Sebastien Jacquemont, Paul Elliott, Tõnu Esko, Anna-Liisa Hartikainen, Jaana Laitinen, Katrin Männik, Danielle Martinet, David Meyre, Matthias Nauck, Claudia Schurmann, Rob Sladek, Gudmar Thorleifsson, Unnur Thorsteinsdóttir, Armand Valsesia, Gerard Waeber, Flore Zufferey, Beverley Balkau, François Pattou, Andres Metspalu, Henry Völzke, Peter Vollenweider, Kári Stefansson, Marjo-Riitta Järvelin, Jacques S. Beckmann, Philippe Froguel, Alexandra I. F. Blakemore

**Affiliations:** 1 Department of Genomics of Common Disease, Imperial College London, London, United Kingdom; 2 Clinical Trial Service Unit and Epidemiological Studies Unit, University of Oxford, Oxford, United Kingdom; 3 Institute for Molecular Bioscience, University of Queensland, Brisbane, Queensland, Australia; 4 Institute of Diagnostics, Clinical Chemistry, University of Oulu, Oulu, Finland; 5 Oulu University Hospital, Oulu, Finland; 6 Department of Epidemiology and Biostatistics, University of California San Francisco, San Francisco, California, United States of America; 7 Service of Medical Genetics, Centre Hospitalier Universitaire Vaudois, Lausanne, Switzerland; 8 Department of Epidemiology and Biostatistics, Imperial College London, London, United Kingdom; 9 MRC Health Protection Agency (HPA) Centre for Environment and Health, Imperial College London, London, United Kingdom; 10 Institute of Molecular and Cell Biology, University of Tartu, Tartu, Estonia; 11 Estonian Genome Center, University of Tartu, Tartu, Estonia; 12 Institute of Clinical Sciences/Obstetrics and Gynecology, University of Oulu, Oulu, Finland; 13 Finnish Institute of Occupational Health, Oulu, Finland; 14 The Center for Integrative Genomics, University of Lausanne, Lausanne, Switzerland; 15 CNRS 8199-Institute of Biology, Pasteur Institute, Lille, France; 16 Department of Clinical Epidemiology and Biostatistics, McMaster University, Hamilton, Ontario, Canada; 17 Institute of Clinical Chemistry and Laboratory Medicine, Ernst-Moritz-Arndt-University, Greifswald, Germany; 18 Interfaculty Institute for Genetics and Functional Genomics, Ernst-Moritz-Arndt-University, Greifswald, Germany; 19 McGill University and Genome Quebec Innovation Centre, Montreal, Canada; 20 Department of Medicine and Human Genetics, McGill University, Montreal, Canada; 21 deCODE Genetics, Reykjavík, Iceland; 22 Faculty of Medicine, University of Iceland, Reykjavik, Iceland; 23 Department of Medical Genetics, University of Lausanne, Lausanne, Switzerland; 24 Swiss Institute of Bioinformatics, University of Lausanne, Lausanne, Switzerland; 25 Ludwig Institute for Cancer Research, University of Lausanne, Lausanne, Switzerland; 26 Department of Internal Medicine, Centre Hospitalier Universitaire Vaudois, Lausanne, Switzerland; 27 INSERM, CESP Centre for Research in Epidemiology and Population Health, U1018, Villejuif, France; 28 University Paris Sud 11, UMRS 1018, Villejuif, France; 29 INSERM U859, Lille, France; 30 Université Lille Nord de France, Centre Hospitalier Universitaire Lille, Lille, France; 31 Institute for Community Medicine, Ernst-Moritz-Arndt-University, Greifswald, Germany; 32 Institute of Health Sciences, University of Oulu, Oulu, Finland; 33 Biocenter Oulu, University of Oulu, Oulu, Finland; 34 Department of Lifecourse and Services, National Institute for Health and Welfare, Oulu, Finland; 35 Section of Investigative Medicine, Imperial College London, London, United Kingdom; University of Bristol, United Kingdom

## Abstract

The limited ability of common variants to account for the genetic contribution to complex disease has prompted searches for rare variants of large effect, to partly explain the ‘missing heritability’. Analyses of genome-wide genotyping data have identified genomic structural variants (GSVs) as a source of such rare causal variants. Recent studies have reported multiple GSV loci associated with risk of obesity. We attempted to replicate these associations by similar analysis of two familial-obesity case-control cohorts and a population cohort, and detected GSVs at 11 out of 18 loci, at frequencies similar to those previously reported. Based on their reported frequencies and effect sizes (OR≥25), we had sufficient statistical power to detect the large majority (80%) of genuine associations at these loci. However, only one obesity association was replicated. Deletion of a 220 kb region on chromosome 16p11.2 has a carrier population frequency of 2×10^−4^ (95% confidence interval [9.6×10^−5^–3.1×10^−4^]); accounts overall for 0.5% [0.19%–0.82%] of severe childhood obesity cases (*P* = 3.8×10^−10^; odds ratio = 25.0 [9.9–60.6]); and results in a mean body mass index (BMI) increase of 5.8 kg.m^−2^ [1.8–10.3] in adults from the general population. We also attempted replication using BMI as a quantitative trait in our population cohort; associations with BMI at or near nominal significance were detected at two further loci near *KIF2B* and within *FOXP2*, but these did not survive correction for multiple testing. These findings emphasise several issues of importance when conducting rare GSV association, including the need for careful cohort selection and replication strategy, accurate GSV identification, and appropriate correction for multiple testing and/or control of false discovery rate. Moreover, they highlight the potential difficulty in replicating rare CNV associations across different populations. Nevertheless, we show that such studies are potentially valuable for the identification of variants making an appreciable contribution to complex disease.

## Introduction

Genome-wide association studies (GWAS) of common single nucleotide polymorphisms (SNPs) have identified loci accounting for only a modest proportion of the heritability of most complex diseases. Although some of this ‘missing heritability’ may be ascribed to a large number of SNPs with weak effect [1], [2], it is becoming increasingly likely that there is a substantial contribution from rare variants with large effect that are not readily identifiable by SNP-based methods [3]–[5]. Thus, resequencing of known risk loci has been pursued to reveal rare point mutations that may have an appreciable impact on disease risk or severity[6]–[8].

We have recently proposed that investigation of genomic structural variants (GSVs) in patients with “extreme” obese phenotypes provides an effective route for the identification of novel obesity-associated loci [9]. Initial reports indicate that subjects with unexplained extreme obesity phenotypes may have a higher aggregate frequency of large GSVs (e.g. >0.5 Mb) compared to the general population [10], [11], strongly suggesting that some of the GSVs carried by these unusual patients are responsible for a pronounced, readily-identifiable phenotype with high penetrance. Genes within the regions delineated by such GSVs may also be of direct relevance to obesity in the general population.

In a first application of this strategy for the identification of novel obesity loci, we showed that a 593 kb deletion on chromosome 16p11.2 (at 29.5–30.1 Mb) directly causes obesity [12]: this association was demonstrated by comparing two cohorts with developmental delay (DD), with or without additional ascertainment for obesity, and was then replicated by retrospective analysis of case-control and population cohorts. We have also shown that duplications of the same locus have the opposite effect, being associated with underweight [13]. Several genes whose altered dosage might plausibly account for the observed phenotype lie within the deleted region, and their potential role in obesity can now be investigated in a hypothesis-driven manner, rather than by the more statistically-challenging hypothesis-free approach applicable to GWAS. Indeed, there are no GWAS signals overlapping this locus [14], [15], illustrating the potential of strategies based on identification of rare GSVs for the identification of novel obesity loci.

A growing number of rare GSVs potentially associated with obesity are now being reported, mainly on the basis of their identification by analysis of GWAS SNP genotyping data. Bochukova, *et al*. [10] compared a small cohort of <300 patients with severe early-onset obesity (half of whom also had developmental delay, DD) with control individuals from the general population, and identified 11 GSV regions that showed association with obesity at nominal significance, including the obesity-associated 593 kb region [12] of chromosome 16p11.2 which is not further studied here. Glessner, *et al.* [16] identified 8 additional GSV loci with nominally significant association with obesity, on the basis of being present in children with “common” obesity (individuals with severe obesity were excluded from the study) but absent from control cohorts of normal weight. The GSVs identified in these two studies vary widely in size, ranging from 2.8 kb to 1.5 Mb, with no overlap between them. With the exception of the independently identified 593 kb deletion of chromosome 16p11.2 [12], [13], all remain to be replicated.

We have attempted to replicate these recently-reported GSV associations with obesity, using algorithmic analysis of genotyping data from obesity case-control and population cohorts. We replicate association with obesity of GSVs at a single locus on chromosome 16p11.2; this locus is distinct from the association on 16p11.2 which we previously reported (using the same cohorts), being separated from it by >600 kb of intervening sequences; the 2 loci are independently associated with obesity. However, we were unable to replicate a high proportion of the remaining regions, and conclude that there is a need for the development and application of robust statistical methods appropriate for testing for association of rare variants from amongst a large collection of GSVs, independent of the platform used for GSV detection. We also highlight the caution required when attempting to support putative associations by phenotyping affected subjects: phenotypic data from a small number of individuals from a highly-selected cohort may not be reliable as an indication of the impact of the variant in unselected subjects.

## Results

### GSV Analysis of Obesity-associated Regions

To investigate the 18 putative associations with obesity reported for rare GSVs [10], [16] (see Table 1), we analysed population and case-control cohorts in a similar manner to that successfully used in our replication of the association with obesity of the 16p11.2 593 kb deletion [12]. Using existing genotyping data from cohorts of severely obese (but with no other reported unrelated health problem) French children and adults, similar numbers of non-overweight controls, and a general population cohort from northern Finland [17], [18], each genomic region was analysed for the presence of GSVs.

**Table 1:** GSV analysis of candidate obesity loci.

	GSV characteristics					child obesitycase-control		adult obesity case-control		Population cohort (NFBC1966)			Replication *P*-value	
	gain/loss	Discovery*P*-value		Size (bp)	Probeson array^a^	non-obese	obese	non-obese	obese	normal	over-weight	obese	case:control	Quantitative trait
Number of samples						557	645	843	701	3126	1617	470		
*Identified in subjects with extreme obesity*														
chr3∶89,250,592–89,319,536	gain	5.07×10^−5^	68,944		3	0	0	0	0	1	0	1	0.491	–
chr6∶52,875,284–52,892,054	loss	1.37×10^−3^	16,770		0	0	0	0	0	0	0	0	–	–
chr8∶143,268,033–143,634,461	gain	1.37×10^−3^	366,428		68	0	0	1	0	1	2	0	*0.509*	0.789
chr10∶541,873–818,440	gain	4.02×10^−3^	276,567		42	0	0	0	0	0	0	0	–	–
chr11∶72,013,333–72,089,312	gain	1.37×10^−3^	75,979		11	0	1	3	0	1	0	0	*0.556*	–
chr11∶105,716,030–106,419,349	loss	3.71×10^−2^	703,319		113	0	0	0	0	0	0	0	–	–
chr15∶28,700,879–30,231,488	gain	7.84×10^−3^	1,530,609		207	0	0	0	0	0	0	0	–	–
chr16∶28,731,428–28,951,376	loss	1.37×10^−3^ b	219,948		28	0	5	0	0	0	3	1	5.48×10^−4^	7.07×10^−3^
chr17∶2,224,814–2,256,880	gain	7.84×10^−3^	32,066		6	0	0	0	0	0	0	0	–	–
chr22∶49,246,176–49,313,898	gain	1.37×10^−3^	67,722		11	0	0	0	0	1	0	0	*0.714*	–
*Identified in subjects with common obesity*														
chr3∶104,059,109–104,092,618	loss	2.21×10^−2^	33,509		2	3	0	0	0	1	1	0	*0.259*	–
chr5∶53,467,427–53,480,255	gain	2.21×10^−2^	12,828		2	0	0	0	0	0	0	0	–	–
chr5∶77,039,051–77,076,628	loss	8.52×10^−3^	37,577		5	1	0	0	2	1	2	0	0.324	0.705
chr5∶83,835,179–83,874,339	loss	3.28×10^−3^	39,160		3	1	0	0	1	0	0	0	0.491	–
chr7∶20,708,193–20,711,088	loss	2.21×10^−2^	2,895		2	1	1	2	0	16	7	4	*0.275*	0.643
chr7∶113,843,696–113,859,679	loss	3.28×10^−3^	15,983		1	0	0	0	1	8	2	0	*0.221*	*4.76*×*10* ^−*2*^
chr17∶49,444,406–49,449,022	gain	8.52×10^−3^	4,616		1	0	0	0	0	3	3	2	0.444	0.103
chr19∶10,489,548–10,512,171	loss	4.87×10^−4^	22,623		4	0	0	0	0	0	0	0	–	–

SNP genotyping data from 3 separate cohorts were analysed using the cnvHap algorithm, and GSVs were identified that corresponded to the GSVs under investigation. For GSVs identified in at least one individual, association with obesity status (excluding overweight individuals from the analysis) was tested according to Fisher’s exact test. For GSVs identified in at least 3 members of the NFBC1966 cohort, association with log_10_BMI as a quantitative trait was tested by 2-way ANOVA with gender as the second covariate. Italics denote a direction of effect opposite to that in the original report.

a refers to the Illumina Human CNV370 array.

b re-calculated after excluding individuals with deletions of the neighbouring 16p11.2 obesity-associated region (see Text S1).

Initial identification of GSVs was carried out using our cnvHap algorithm, which is applicable to data from a wide range of platforms (including Illumina and Affymetrix genotyping arrays, CGH arrays and next-generation sequencing), and which has greatly improved sensitivity and specificity for detection of short GSVs compared to other commonly-used algorithms [19]. To ensure that our analysis mirrored the procedures that led to the original reported associations, we scored only those GSVs that were of a similar type (deletion/duplication) and that spanned the entirety of the GSV region. In addition, to ensure that only high-confidence calls were included, for the shorter candidate regions (those for which we had probe coverage of 6 or fewer probes – see Table 1) we required that a GSV call included a minimum of 3 consecutive probes in all cases, irrespective of the size of the region being analysed.

**Figure 1:**
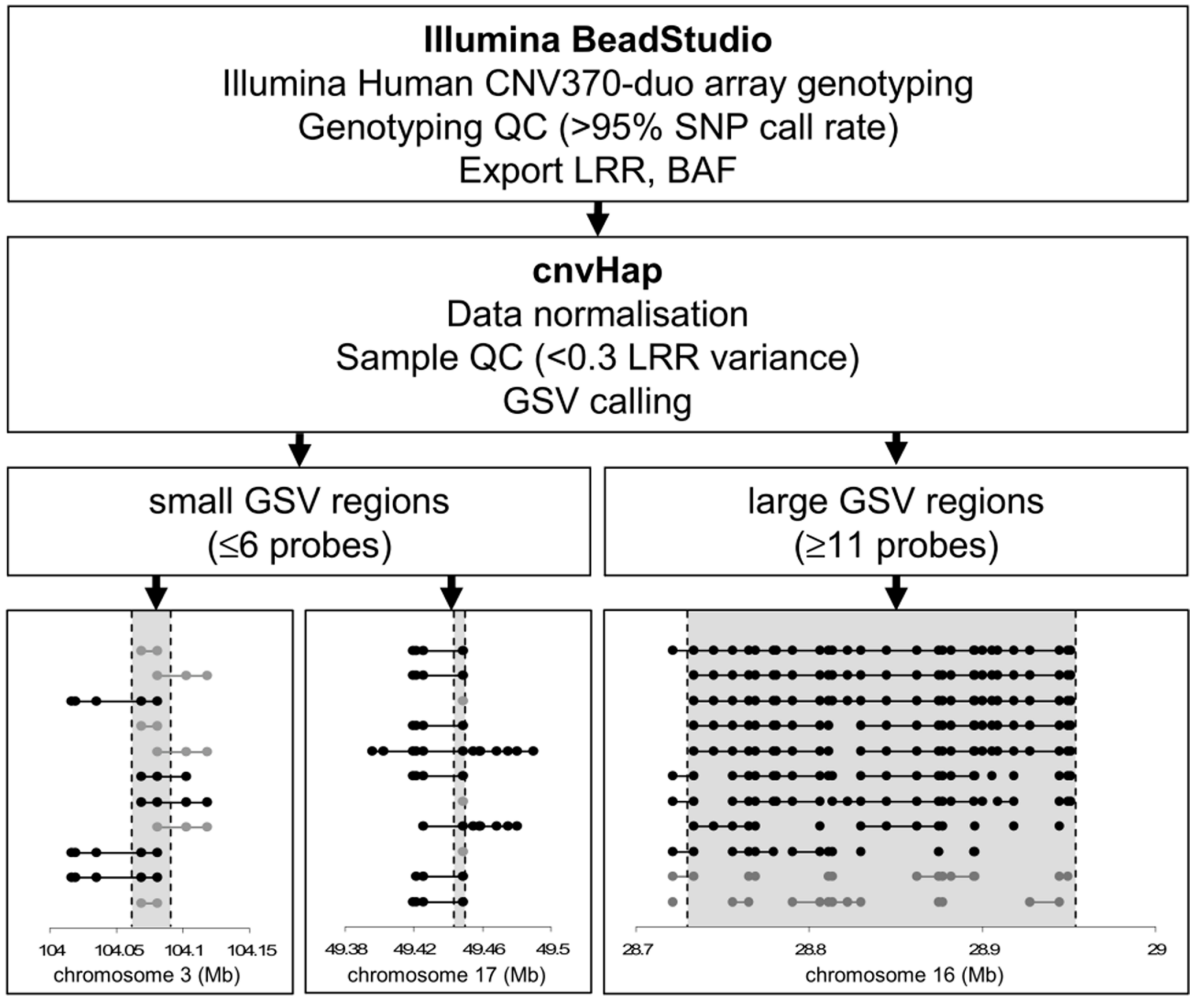
Procedure for identification of GSVs. Following data export and QC, GSV calling was carried out using the cnvHap algorithm. Illustrative data for 3 GSV loci (shaded) show all positive GSV calls (black) together with examples of calls not meeting the necessary criteria (grey); probes at which copy number changes were identified are also indicated (circles).

This procedure was applied to our cohorts for each of the 18 loci under investigation (8 identified in subjects with common obesity [16], 10 in subjects with extreme obesity [10]). Consistent with the original reports, short GSV regions often featured multiple overlapping aberrations with varying lengths and breakpoints (see Figure 1); by contrast, aberrations identified for larger GSV regions were much more consistent in both size and breakpoint location. The results of the analysis, summarised in Table 1, revealed somewhat different patterns of occurrence for the 2 sets of GSVs. For the 8 GSVs originally identified in subjects with common obesity, the overall frequency of calls at these loci in our cohorts (63 calls in a total of 7959 subjects) was 25% higher than that in the original report (42/6634), and GSVs at 6 out of 8 loci were detected at least twice. By contrast, the number of calls (21 in total) for the 10 extreme obesity GSV loci represented a 30% lower frequency than in the original report (29/7650), and only 5 out of 10 were detected at all: It was notable that the 5 detected were those originally identified only in subjects either lacking DD or with only mild DD. The remaining 5 were originally identified in subjects with pronounced DD [10], raising the possibility that they were not detected because subjects carrying them tended not to be recruited to our cohorts.

We also investigated the occurrence of reciprocal GSVs at each locus (i.e. duplications instead of deletions and *vice versa*), applying the same calling criteria (Table S1). Although the overall frequency of reciprocal GSVs was slightly lower compared to those showing reported association with obesity, there was a difference between the two sets of loci: Common obesity GSVs were identified approximately twice as frequently as their reciprocal counterparts, but extreme obesity GSVs were 30% less frequent than reciprocal aberrations. For three GSV loci in particular (those on chromosomes 3, 17 and 22), all originally identified in subjects with DD, there was a clearly higher frequency of the reciprocal event in population cohorts; this is again consistent with the GSVs identified only in DD patients having reduced prevalence in the general population.

### Case-control Replication Analysis

Combining subjects from the population cohort who were obese (BMI ≥30 kg.m^−2^) or normal weight/underweight (BMI <25 kg.m^−2^) with the corresponding case-control subjects, and assuming a GSV has a dominant effect, the combined cohort was sufficient to give >98% power to detect associations (at *P*<0.05 for Fisher’s exact test) of GSVs present almost exclusively in obese subjects (odds ratio, OR = 50) at a frequency in cases of 0.005, or with power of 94% or 83% for odds ratios of 10 or 5 respectively; even for a GSV frequency in cases of 0.002, power was 67% (OR = 50), 53% (OR = 10) or 40% (OR = 5). On the basis of the observed GSV frequencies and ORs in the original reports, median power was 79.8% (minimum 53%) for the 11 loci for which the corresponding GSVs were detected in our cohorts; thus, we might expect to replicate ∼80% of genuine associations. Although this is likely to be something of an overestimate because of OR overestimation due to the “winner’s curse” [20], the minimum OR in the original reports was 25 [10], [16]), and even for much lower effect sizes with OR≥5 we would nevertheless expect to detect 62% of associations (71% of those with OR≥10).

Although GSVs at these loci were observed at similar overall frequencies to those in the original reports, we observed a low rate of replication for associations with obesity (Table 1). For 10 of the 11 GSVs detected, the reported obesity association was not replicated, even at nominal levels of significance. Of particular note was that each of the 6 GSVs originally identified in subjects with common obesity was present in at least 1 normal weight or underweight individual, contrary to the criteria used to identify these GSVs (i.e. being present exclusively in obese subjects) [16]. Indeed, for the majority of loci the GSV frequency was higher in normal weight than in obese subjects.

For a single GSV, however, the association with obesity was strongly replicated. A deletion of 220 kb on chromosome 16p11.2 (at 28.73–28.95 Mb) affecting several genes including *SH2B1*, was identified in 6 obese individuals compared to none in normal weight subjects (*P* = 5.48×10^−4^). This deletion spans a locus implicated in obesity in SNP-association studies [14]. Of note, apart from rare instances of more extensive deletions spanning both regions [10], [21], which complicated the previous analysis of this region (see Text S1), this 220 kb region is completely separate from the 593 kb locus (also on 16p11.2) whose association with obesity/underweight has been previously reported by us [12], [13]; they are 600 kb apart, there is no discernible linkage disequilibrium between SNPs within each region (Figure S1), and copy-number changes at the 593 kb locus have no consistent effect on expression of genes at the 220 kb locus [13]. Thus, each locus is independently associated with obesity.

### The Contribution of Chromosome 16p11.2 220 kb Deletions to Obesity

Consistent with the original report for the *SH2B1* locus [10], 5 of the affected subjects were from our cohort of severely obese children, a significant enrichment compared to our general population cohort (*P* = 1.4×10^−3^). Extending the analysis to include multiple other population cohorts (Table 2) unambiguously confirmed the association between this deletion and childhood obesity (*P* = 8.7×10**^−^** ^7^; OR = 38.4, [95% confidence interval = 10.4–120.6]). This finding was further strengthened (*P* = 3.8×10**^−^** ^10^, OR = 25.0 [9.9–60.6]) by inclusion of previously published data [10], [21].

**Table 2:** Replication of obesity association for 220 kb deletion on chromosome 16p11.2.

Cohort	Deletions	Total	*P*
***Child obesity***			
** Child obesity (France)** ^a^	**5**	**645**	**8.74×10^−7 a^**
*Published data*			
Severe early-onset obesity (UK)	3	278	
GOOS (UK)	2	1,062	
** CHILD OBESITY TOTAL** ^b,c^	**10**	**1,985**	**3.81×10^−10 b^**
***Adult obesity***			
Adult obesity (France)	0	701	
Bariatric weight-loss surgery (France)	0	139	
** ADULT OBESITY TOTAL** ^c^	**0**	**840**	**0.039^ c^**
***General population***			
NFBC1966 (Finland)	4	5,213	
EGCUT (Estonia)	0	2,665	
CoLaus (Switzerland)	1	5,612	
deCODE (Iceland)	6	36,583	
SHIP (Germany)	0	4,068	
** TOTAL** ^a^	**11**	**54,141**	
*Published data*			
WTCCC2/GAIN (UK/US)	2	7,362	
ISC/PARC/NINDS/HGDP/CHOP (Europe/US)	1	7,700	
** POPULATION TOTAL** ^b^	**14**	**69,203**	

Instances of the 220 kb deletion were identified in multiple cohorts by analysis of SNP genotyping data, with subsequent validation by MLPA or qPCR. Published data were as according to the respective reports [10], [21].

a,b,c Differences between pairs of combined cohorts, as indicated, were tested using Fisher’s exact test.

Intriguingly, the association with adult obesity is less clear. We investigated by MLPA the parents of the 5 severely obese children carrying the deletion, finding that 4 deletions were inherited (one arising *de novo*). However, only two of the four adult carriers were obese and there was no significant difference in BMI between the carrier and non-carrier parents (*P* = 0.15, Student’s *t*-test). Furthermore, not only were no deletions identified in a total of 840 subjects from adult severe obesity cohorts (a significant difference from the overall frequency for child obesity, *P* = 0.039), but out of 8 adult carriers from our population cohorts, only 3 were obese. Nevertheless, a further 4 were overweight (BMI ≥25 kg.m^−2^) so that, overall, adult carriers had a mean BMI of 30.2 kg.m^−2^ [27.3–33.3], with a mean Z-score (relative to their respective population distributions) of +1.10 [+0.34–+1.86] (*P* = 9.14×10^−4^, one-tailed *Z*-test). The impact of the deletion in terms of BMI is made clear from comparison of the 4 carriers from NFBC1966 and the remainder of this cohort (mean BMI change = +5.8 kg.m^−2^ [+1.5–+10.8]; *P* = 3.53×10^−3^, one-tailed *t*-test). Thus, adult carriers of this deletion show an appreciable increase in BMI, but this is not necessarily sufficient for them to cross the threshold into clinical obesity.

**Figure 2:**
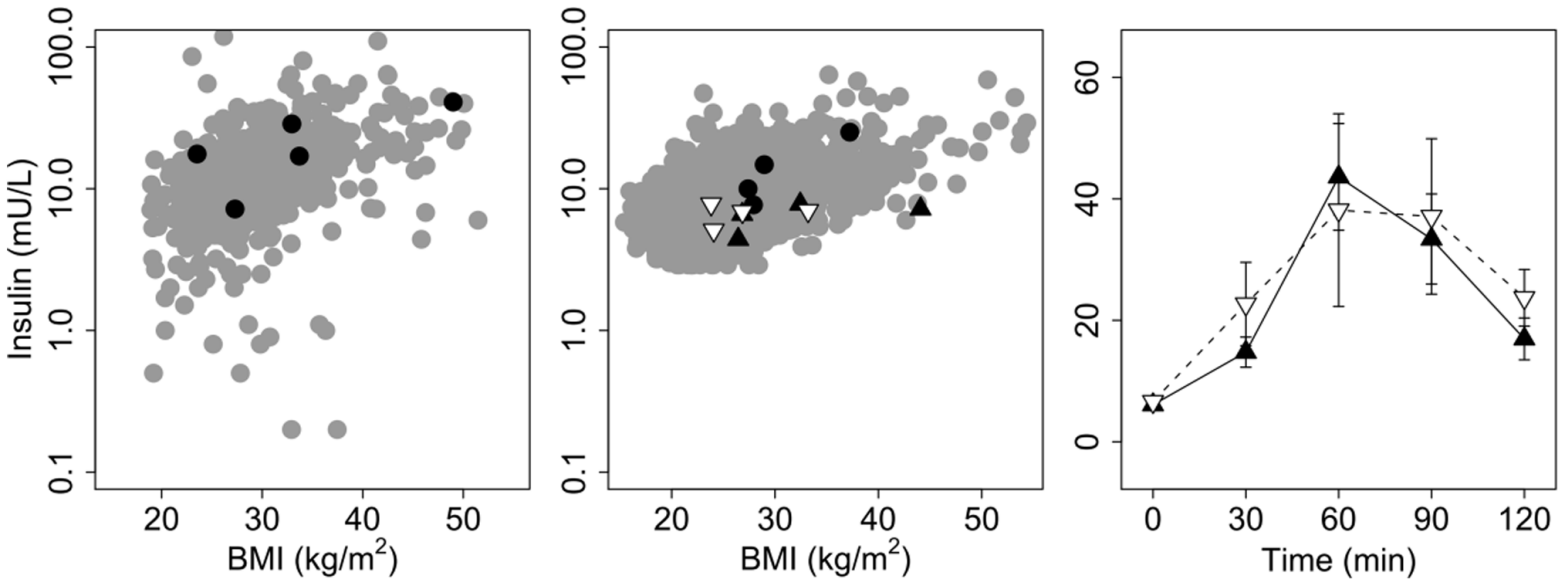
Metabolic phenotype of carriers of a 220 kb deletion at chromosome 16p11.2. **(a)** Fasting plasma insulin levels relative to BMI, for 558 normoglycaemic severely obese children from northern France either carrying a deletion (black) or not (grey). **(b)** Fasting plasma insulin levels relative to BMI, for 5254 normoglycaemic 31 year-old Finns either carrying a deletion (black circles) or not (grey circles). Also shown are the parents of obese French child probands who carry a deletion (black triangles) or not (white triangles). **(c)** Plasma insulin levels in response to a 75 g oral glucose load in parents of obese child probands. Data shown are mean ± SEM for carrier parents (n = 3, mean BMI = 28.6 kg.m^−2^, black triangle) and unaffected parents (n = 4, mean BMI = 27.0 kg.m^−2^, white triangles).

The original association between this GSV and obesity was supported by reported disproportionate extreme hyperinsulinaemia in carriers of the deletion [10]. We sought to confirm this finding by investigating fasting insulin and the response to oral glucose in the subjects from our study. However, we found no evidence in our cohorts for the reported phenotype. Compared to the remainder of the cohort from which they were drawn, levels of fasting insulin in carriers of the deletion were not discernibly different from those expected for a subject’s BMI, for both children (Fig. 2a) and adults (Fig. 2b), with no indication of the reported 3-fold increase; similar conclusions were drawn when the comparison was limited to individuals of the same gender and age (Figure S2). Equally, no difference was observed in either fasting insulin (Fig. 2b) or in the insulin response to oral glucose (Fig. 2c) between carrier and non-carrier parents of child probands.

### Quantitative Trait Replication Analysis

As noted above, a significant association with obesity of deletions of the *SH2B1* locus was identified by quantitative analysis of BMI in the NFBC1966 population cohort alone, Thus, the reduced sample size was compensated for by inclusion of subjects with intermediate phenotypes (i.e. overweight) and the increase in statistical power that derives from analysis of quantitative traits compared to case-control approaches to association testing; indeed, this advantage becomes progressively more marked at lower allele frequencies for the genetic marker under test [22]. Therefore, we investigated whether any other putative GSV-obesity associations were replicated when using this approach. For each candidate GSV that was identified in at least 3 NFBC1966 subjects – the *SH2B1* locus and 5 other loci (Table 1) – and also the previously-identified 16p11.2 obesity locus, we conducted a 2-way analysis of variance, with gender and GSV status as explanatory factors and log-transformed BMI as the response variable. Since several individuals carried more than one of these GSVs, we also conducted a single, combined, multifactorial analysis of these 7 GSVs; this gave very similar results to those for the separate individual tests. Alternative approaches (e.g. 2-tailed heteroscedastic *t*-tests using gender-corrected BMI data) also yielded similar results.

Three loci tested did not give significant association with BMI although, for two of these, statistical power was limited because only 3 carriers were identified – this number of carriers permits only moderate significance (*P* = 7.45×110^−3^) even for the 593 kb deletions of chromosome 16p11.2 that are known to be strongly associated with morbid obesity [12], [13]. However, in contrast to the case-control replication analysis, this quantitative approach provided limited evidence to support involvement in BMI of 2 loci (in addition to the confirmed association with deletions in the *SH2B1* region), albeit only at or near nominal significance insufficient to survive correction for multiple-testing. There was suggestive evidence for association with BMI of duplications near to the *KIF2B* gene on chromosome 17q22 (*P* = 0.103; mean BMI change = +2.3 kg.m^−2^ [–0.4 – +5.4]); and deletions at a second locus within the *FOXP2* gene on chromosome 7q31.1 were nominally associated with reduced BMI (*P* = 0.0476; mean BMI change = –2.3 kg.m^−2^ [–4.4 – –0.03]). However, this latter effect was opposite to the increased risk of obesity originally reported [16].

**Figure 3:**
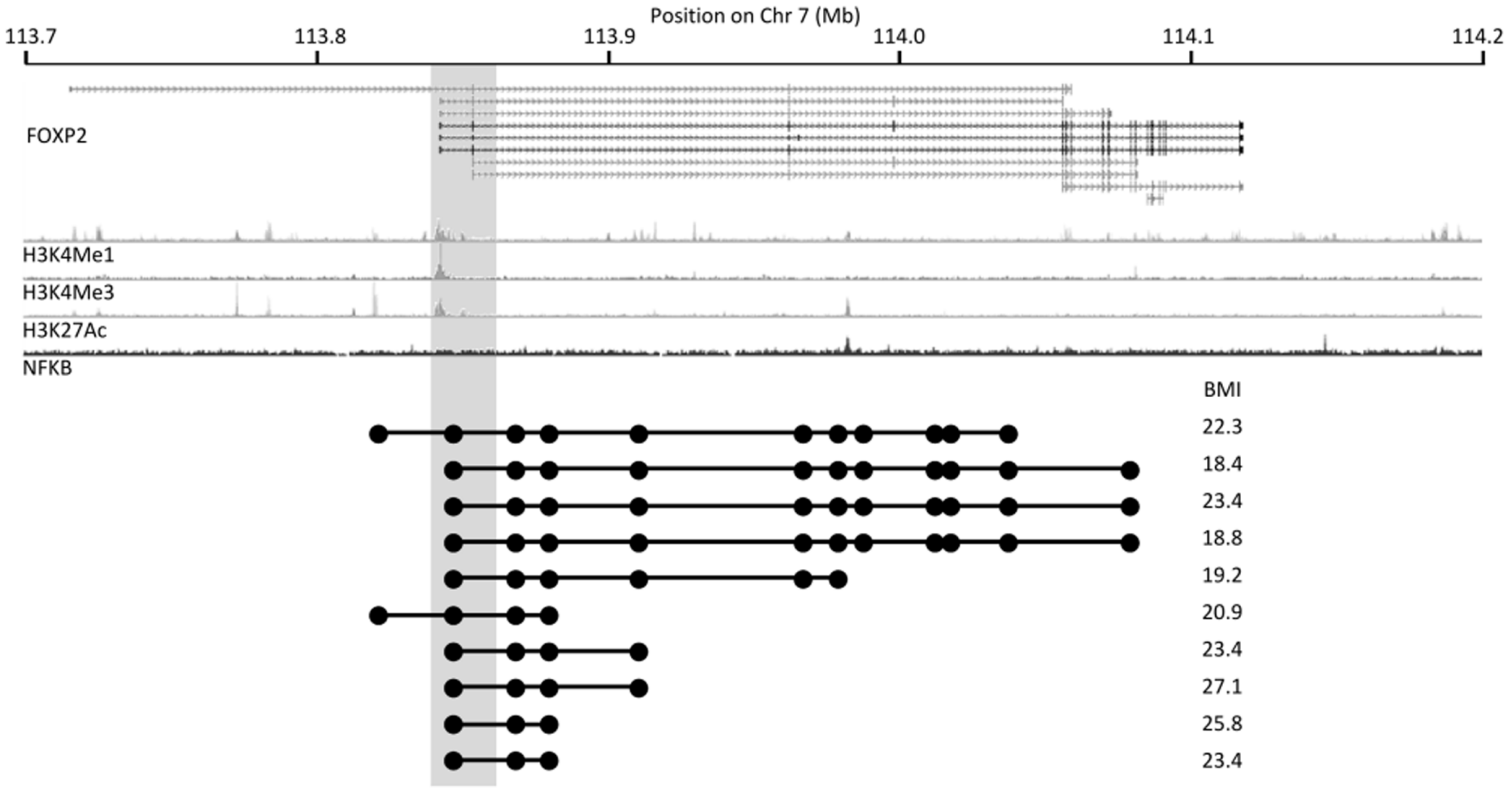
Reduced BMI in carriers of deletions in the *FOXP2* region. Deletions within *FOXP2* are shown relative to selected tracks from the UCSC browser (http://genome.ucsc.edu) for the corresponding region of chromosome 7: *FOXP2* coding transcripts (UCSC Genes); histone modifications H3K4Me1, H3K4Me3, H3K27Ac (ENCODE Regulation); and binding by transcription factor NF-κB (ENCODE TFBS). Multiple additional transcription factors bind at the apparent NF-κB binding site. The minimum extent of each predicted deletion, the probes at which copy number changes were identified and the BMI for carriers of each deletion are as shown. Grey shading indicates the region previously associated with BMI [16].

To investigate these loci in more detail, we assessed the potential functional impact of the individual GSVs carried by these individuals. For duplications on chromosome 17q22, all GSVs identified in our study affected intergenic sequences and covered the same genomic region as was spanned by the GSVs previously reported as associated with obesity. However, of the 10 predicted deletions at the locus on chromosome 7q31.1 that were identified in our population cohort, 5 extend substantially beyond the GSV region previously identified as obesity-associated (Figure 3), which spans 1–3 small exons that (depending on the splice variant) encode either part of the 5′-untranslated region of the *FOXP2* mRNA or a small part of the N-terminal of the protein. By contrast, the larger deletions identified in our analysis are predicted to include several additional exons and also a possible binding site (as indicated by ChIP-seq) for transcription factors including NF-κB, which has been implicated in the regulation of adipocyte differentiation and proliferation [23]. Thus, these larger variants may have very different functional effects from the smaller deletions. Consistent with this, the subjects carrying the 5 largest putative deletions in this region had significantly reduced BMI compared to both the population (*P* = 7.8×10^−3^, mean BMI change = –4.2 kg.m^−2^ [–6.8––1.2]) and carriers of the smaller deletions (*P* = 0.0177, one-tailed *t*-test). The smaller variants had no discernible impact on BMI (*P* = 0.88).

## Discussion

The analysis of rare GSVs for association with complex traits represents a complementary approach to SNP- or sequence-based methods for identifying novel loci that can account for the ‘missing heritability’ of multiple complex traits [3], [9]. Even though causal GSVs themselves may be rare and found only in individuals with extreme phenotypes, the identification of such GSVs can enable a more focussed search for rare causal sequence variants. This logic lay behind the elucidation of the impact on obesity of defects in *SIM1*. The original identification of *SIM1* as a possible obesity gene was as a result of its disruption due to a chromosomal rearrangement (a balanced translocation) in a single individual with profound obesity [24]; this was followed by the identification, by exon sequencing, of rare *SIM1* variants that co-segregate with syndromic obesity and of common variants implicated in common obesity [25], [26]. The potential of this approach to reveal additional novel obesity-associated loci is supported by our analysis, which provides evidence to support reported GSV associations at 3 loci [10], [16].

Despite being well-powered to confirm the majority of true associations, and identifying GSVs at similar overall frequencies to the original reports, only one reported association was confirmed using a case-control approach. We also conducted tests for association with BMI as a quantitative trait, for those loci at which GSVs were identified sufficiently frequently in our population cohort (for which there was no prior ascertainment on the basis of obesity). Of 3 GSVs present in >0.1% of subjects, 2 showed association with changes in BMI at or near nominal significance. Duplications of a region lying between the *KIF2B* and *TOM1L1* genes showed marginal association with increased BMI, consistent with the original report [16]; there is at present no readily apparent functional basis for this putative association. Intriguingly, the second nominally-significant association was between deletions within the *FOXP2* gene and decreased BMI, an effect in the opposite direction to that previously reported for the locus. This apparent directional inconsistency is likely to be due to the influence of GSVs that are appreciably larger than those previously reported, suggesting that the different variants identified at this locus have widely varying functional effects. A role for *FOXP2* in obesity is supported by the presence within the gene of independent SNP associations at *P*<10^−3^ with all of BMI [14], waist-hip ratio (adjusted for BMI) [15] and insulin resistance [27] (Figure S3). A plausible basis for association between *FOXP2* variants and obesity is through its involvement in neurodevelopment [28], whose importance in feeding behaviour is well-established [29]; alternatively, an obesity-related phenotype might be independent of effects on *FOXP2*, and result instead from deletion of a putative NF-κB binding site.

Although the 2 associations above provide tentative support for the original reports that these loci may play a role in obesity, they are nevertheless insufficient to survive correction for multiple testing. The only association unambiguously replicated by our study was that between a 220 kb deletion of chromosome 16p11.2 and obesity. It is interesting to note that this second replicated locus lies only 600 kb from that previously identified, also on chromosome 16p11.2, and that both deletions arise *de novo* with high frequency, probably reflecting general chromosomal instability on chromosome 16p due to the presence of multiple segmental duplications [10], [12], [21]. The high rate of recurrence of these deletions likely contributed to their early discovery and replication using these methods.

We confirm a marked increase in the risk of severe childhood obesity in carriers of the 220 kb deletion, which accounts for a total of 0.5% of the combined cases from our study and the original report. The impact on obesity status in adult carriers appears less pronounced, but there is nevertheless an appreciable increase in BMI (corresponding to 15–19 kg in weight for subjects 160–180 cm in height). There are several possible reasons for the apparent difference between children and adults: it may reflect population differences (the child carriers of the GSV were from France and the UK, the adults primarily from Nordic countries); it may reflect cohort ascertainment, for instance that the child cohorts did not include overweight or mildly obese subjects – it is notable, however, that the deletion was not reported at a comparable frequency in cohorts of children with common obesity [16]; it may reflect a genuine attenuation of the effect of the deletion in adults, so that impact of the GSV on obesity becomes less pronounced with increasing age; or the severe obesity observed in children may have been triggered by an aspect of the modern obesogenic environment that was experienced to a lesser degree by older subjects.

The reported disproportionate hyperinsulinaemia in carriers of these deletions is reminiscent of the phenotype of *SH2B1* knockout mice [30], which previously led to the suggestion that haploinsufficiency of *SH2B1* is the primary cause of obesity in these individuals [10]. However, the absence of evidence in our data to support this phenotype reopens the possibility that haploinsufficiency of one of the other genes in the region is responsible for the observed GSV-associated obesity phenotype (although *SH2B1* remains a strong candidate). It also highlights the caution required when interpreting data derived from a heavily-selected cohort – carriers of a GSV drawn from such a cohort do not necessarily accurately reflect the phenotypic effect of a GSV in the general population. We suggest that, if possible, attempts to investigate additional phenotypes that may be associated with a variant should not use individuals that have been ascertained on the basis of the phenotype of interest; instead, they should be drawn from independent cohorts and selected solely on the basis of being a carrier of the variant under study.

There are many issues that remain to be addressed when seeking to identify GSVs that are associated with complex disease, several of which are highlighted by this study; some of these issues are also relevant to the analysis of rare variants identified by sequencing approaches [31], [32]. In particular, we make the following observations and recommendations:

### Cohort Selection

Ascertainment according to broad criteria can give only limited enrichment of rare variants. Conversely, cohorts selected on the basis of pronounced phenotypes may be difficult to recruit, a potential problem not only for variant discovery but also for replication, especially where the discovery cohort includes individuals not normally recruited to other cohorts (e.g. those with DD). For instance, we were unable to attempt replication of GSVs that were originally identified in cases with severe obesity plus DD since very few of these were detected in our cohorts; some of these may reflect novel ‘syndromic’ forms of obesity whose replication will require analysis of additional ‘obesity plus’ cohorts. We also note that recruitment of cohorts of sufficient size by including subjects from a range of localities or ethnicities may introduce complications, since the majority of population groups, both within Europe and worldwide, have not yet been assessed for population specific GSVs. This also poses a problem for replication – the failure to detect some GSVs in our cohorts may reflect low frequencies in the populations from which they were drawn. Furthermore, although subjects carrying a highly-penetrant causative variant might naïvely be expected to display the phenotype regardless of ethnicity, geographical origin or environmental exposure, we have very little information on the potential for cohort-specific modifiers that can confound that expectation, or on differences in the frequencies or characteristics of rare variants between different populations. As a minimum, therefore, it is essential to include appropriate geographically-matched controls (as was the case in the original reports that are the subject of our replication study).

### GSV Detection

In general, large GSVs are more readily detected from genotyping or CGH array data, but occur infrequently so that phenotype associations are difficult to demonstrate statistically. Conversely, accurate calling of smaller GSVs spanning only a few probes remains problematic, despite ongoing improvements in methods for GSV detection [19], [33], [34]. Inaccuracy in GSV calling, with an appreciable frequency of both false positive and false negative calls, results in inflated *P*-values and an increased rate of false-positive associations (Text S2; Figure S4). This is conceptually equivalent to other scenarios in which genome-wide inflation occurs as a result of genotyping inaccuracy [35] – indeed, attempts to apply algorithmic detection of GSVs to genome-wide genotyping data yield results with marked inflation [36] (our unpublished observations) – and we suggest that the appropriate correction is to apply established methods of genomic control, e.g. scaling of χ^2^ values according to the genomic inflation parameter λ.

### Overlapping GSVs and Variable Effects

Although different instances of large GSVs commonly have approximately the same boundaries [10], with a correspondingly high probability of having the same or similar phenotypic effects, smaller GSV regions routinely feature a range of overlapping GSV calls of different sizes and locations [36], [37], thereby presenting a dilemma – in the absence of strong prior information to enable modelling of the effects of different GSV variants, how should a range of variants affecting a single locus be combined when testing for association with a phenotype of interest? One approach, analogous to methods such as the ‘cohort allelic sums test’ [38] developed for analysis of multiple rare sequence variants within a gene, is to treat a set of overlapping GSVs as functionally identical, effectively discounting the structural complexity, so that only a single hypothesis related to a putative functional effect is tested. Although perhaps appropriate where, for instance, all variants are predicted to have similar functional consequences due to directly disrupting or deleting a particular gene or due to affecting an intergenic region, this approach requires user intervention and is not universally applicable (e.g. in the context of a genome-wide analysis). A more general unsupervised approach for analysis of such complex loci, used in the studies examined here [10], [16] and also in analyses of common GSVs [36], [37], [39], is to test separately at multiple probe locations within a region. However, as illustrated by our analysis of the *FOXP2* locus, it cannot be assumed that overlapping but distinct variants have similar effect sizes or even directions; furthermore, such ‘point-wise’ analysis entails multiple statistical tests at each GSV locus, leading to potential inflation in the reported *P*-value for the region as a whole. Thus, there is a need for methods that properly address the structural complexity frequently observed at GSV loci. One straightforward approach might be to apply a locus-specific multiple-testing correction, according to GSV complexity, to reflect the number of independent tests made at a locus, in a manner similar to that used to correct for multiple tests of SNPs in linkage disequilibrium [40]. Alternatively, more sophisticated methods developed for aggregating rare sequence variants in the absence of prior information, for instance as implemented in the ‘thgenetics’ R package [41], might be adapted for use with complex GSVs.

Although problematic for the identification of an association, we nevertheless note that the existence of multiple different GSVs may be of great utility in dissecting a locus whose association has been conclusively demonstrated.

### Statistical Power and False Discovery

Even after enrichment by selection of an appropriate discovery cohort, and testing only for rare GSVs with dominant phenotypic effects, low statistical power remains an important issue, with increased rates of false negative and false positive associations at a given significance threshold and inflated estimates of effect size (“winner’s curse”) [20], [31], [32]. As noted above, increasing cohort size to give improved power and/or reduced type I error rate may not be readily achievable for highly specific phenotypes. A second statistical issue, common to all scans for variant associations, is that each of the many GSVs detected in even a small cohort is the subject of a separate independent hypothesis, so that substantial multiple testing correction of the significance threshold or rigorous control of the false discovery rate (FDR) is required if a large type I error rate is to be avoided. Even after the use of predefined criteria to select a subset of GSVs for analysis, the necessary correction remains substantial: In the previous studies, subjects with common obesity carried approximately 20 GSV calls per individual (1080 cases, 2500 controls) [16]; more than 300 separate rare GSV loci were identified in the extreme obesity discovery cohort alone [10]; and both used a point-wise method for assessing association at complex GSV regions. Although attempts were made to take account of multiple testing (e.g. exclude GSVs present in the Database of Genomic Variants, including only rare GSVs, empirical estimate of FDR), it is unclear to what extent these methods are effective – methods developed for association analysis of rare sequence variants are not consistently well-powered even for large sample sizes [42]. Indeed, inspection of the original overall *P*-values for each GSV (Table 1: reproduced from or calculated according to the original reports; see Text S1) shows that even a moderate correction to the threshold for significance to account for these multiple tests would have excluded the majority of the reported loci. Thus, our observation of an apparent high rate of false positives is likely to reflect insufficient control of the FDR.

We suggest that a potentially useful approach is to adopt a two-stage study design: initial genome-wide analysis of case-control cohorts for GSV discovery, although likely to be underpowered, will nevertheless yield a set of candidate GSVs; unselected population cohorts can then be screened for individuals carrying these GSVs. The key advantage of this approach is that carriers identified from population cohorts are not biased (either qualitatively or quantitatively) by pre-existing ascertainment criteria, so that the impact of the GSV on phenotype can be directly analysed using more powerful quantitative methods.

Our replication of the obesity association of deletions including *SH2B1* and the finding of limited evidence to support 2 further associations, together with recent successes in other disorders including attention deficit hyperactivity disorder [43], demonstrate that analysis of carefully-selected cohorts has the potential to reveal novel, rare, causal GSVs. However, it is clear that there remains a need for an accepted foundation on which to base genome-wide searches for rare variants. In its absence, attempts to overcome the unavoidable lack of statistical power may lead to the adoption of methods whose effectiveness is not readily quantifiable. Thus, there is a danger that reported associations may include a large number of false positives. Similar caveats should perhaps also be attached to the growing number of studies investigating common GSVs [36], [37], [39]. Although careful experimental design and the inclusion of additional phenotypic and/or experimental data can help to limit this problem, our findings illustrate the urgent need for well-defined, robust statistical methods that are readily applicable to the search for causal, rare, genomic structural variants.

## Materials and Methods

### Cohorts

Initial replication analysis was of cohorts used in our previous work [12], with ethnic outliers removed as described. Obesity case-control cohorts from France were as previously published [17]: Phenotypes and genotyping data (Illumina Human CNV370-duo arrays) passing quality control were available for 649 obese children with a body mass index (BMI) ≥97^th^ percentile corrected for gender and age; 557 non-obese controls (BMI ≤90^th^ percentile); 705 obese adults (BMI ≥40 kg.m^−2^) and 843 non-obese controls (<25 kg.m^−2^). Data for 141 severely obese French patients undergoing elective bariatric weight-loss surgery were as previously described [12]. For *The Northern Finland Birth Cohort 1966* (NFBC1966) prospective birth cohort [18], phenotypic data and genotyping data (Illumina Human CNV370-duo arrays) passing quality control was available for 5,216 subjects aged 31 years at the time of phenotyping. For further replication of the 220 kb deletion on chromosome 16p11.2, genotyping data was available for other previously-described population cohorts as follows: the *CoLaus* prospective population cohort [44] –5,612 white individuals aged 35–75 years randomly selected from the general population in Lausanne, Switzerland; the *EGCUT* BioBank [45] –2,666 individuals randomly selected from the 48,000 Estonian participants; the *deCODE* population cohort [46]–36,601 recruited from the whole of Iceland; the *SHIP* cross-sectional survey cohort [47], [48] –4,070 German citizens from Western Pomerania. In all cases, individuals in the above cohorts were excluded from the analysis if they had previously been shown to carry single-locus obesity variants (e.g. in *MC4R*); specifically excluded were those carrying the obesity-causing deletion of 593 kb on chromosome 16p11.2 (4 child obesity, 4 adult obesity, 2 bariatric patients, 3 NFBC1966, 1 EGCUT, 18 deCODE, 2 SHIP) [12]; no subject was related to any other subject. All participants or their legal guardians gave written informed consent, and all local ethics committees approved the study protocol. EGCUT is conducted according to Estonian Gene Research Act. For deCODE, all procedures related to this study have been approved by the Data Protection Authority and National Bioethics Committee of Iceland.

### GSV Identification

The GSV regions were selected either as stated in the original report [16] or the region common to all GSVs identified in that region [10], and were analysed according to the GSV analysis pipeline illustrated in Figure 1. Intensity data from the French and NFBC1966 cohorts were exported from Illumina BeadStudio in the form of logR ratio (LRR) and B Allele Frequency (BAF); samples with a low SNP call rate (<95%) or a genome-wide LRR variance >0.3 were excluded. The cnvHap algorithm with default parameter settings (false discovery rate ∼5%) was applied to each region under investigation plus additional 500 kbp flanking regions; using these parameters we expect high sensitivity for GSV detection – even a false discovery rate as low as 1% gives genome-wide sensitivity of ∼40% for GSV detection in an individual, and >60% for identifying the presence of a GSV in a cohort [19]. The initial (unsupervised) GSV detection was further improved by a series of manual procedures applied to each GSV locus under study: Only GSV calls covering at least 3 consecutive probes were considered; for short GSV regions spanning 6 or fewer probes, GSV calls were required to span the entire region; and SNP cluster plots were manually inspected to confirm both positive and negative GSV calls and to check for possible artefactual sources of differential GSV detection between cases and controls. For longer GSV regions (i.e. spanning ≥11 probes), it was also necessary to manage the effects of data noise or of the presence of a second small GSV in the same location on the homologous chromosome on GSV calling; a side-effect of the improved sensitivity of cnvHap is that, particularly for samples with lower data quality, larger GSVs are sometimes split into several smaller GSV calls. Thus, a modified procedure was employed: GSV calls across the entire region were combined, and individuals with copy number changes (i.e. deletion or duplication, as appropriate) at over 50% of probes within the region were provisionally called as carrying a GSV; the presence of a full-length GSV in these individuals was then confirmed by manual inspection of the LRR and BAF data. This approach again minimises the potential for artefactual associations arising from different GSV call-rates in cases and controls (e.g. due to differences in DNA quality). Furthermore, for both adult and child case-control cohorts, any potential bias in GSV detection favoured an increased call rate in cases (higher mean number of GSV calls per subject) which would be expected to favour false positive associations and to mitigate against false negatives. All chromosomal coordinates are given according to genome build 36 (hg18).

Screening for the 220 kb deletion on chromosome 16p11.2 in GWAS data from additional cohorts was variously carried out using a Gaussian Mixture Model [49]; Circular Binary Segmentation [50], [51]; QuantiSNP [52]; PennCNV [53]; BeadStudio GT module (Illumina Inc); and Birdseed [54]. At least two independent methods were used for each cohort. Where DNA was available, GSV calls at this locus were confirmed and probands’ parents investigated by multiplex ligation-dependent probe amplification [55], using the oligonucleotide probe set previously described [10] (kind gift of I.S. Farooqi).

### Fasting Insulin and Oral Glucose Tolerance test

Data for insulin, after fasting and following 75 g oral glucose, were from previously-reported studies [18], [56]. Plasma insulin was assayed by radioimmunoassay (Pharmacia Diagnostics) in blood samples drawn either after overnight fasting or at 0, 30, 60, 90, and 120 min after glucose ingestion.

### Statistics

Tests for case-control association and calculation of odds ratios were carried out using the fisher.test function, tests for differences in log-transformed BMI used the analysis of variance aov function, and *Z*-test for deviation from population mean used the Student’s *t*-test t.test function, each as implemented in R [57]. Calculations of post-hoc achieved power for one-tailed Fisher’s exact test were carried out using G*Power version 3.1.2 [58].

## Supporting Information

Figure S1 **Linkage disequilibrium in the chromosome 16p11.2 region.**
(PDF)

Figure S2 **Fasting plasma insulin in obese children stratified by age and gender.**
(PDF)

Figure S3 **SNP associations in the** ***FOXP2*** **region.**
(PDF)

Figure S4 **Impact of GSV call inaccuracy on type I error rate.**
(PDF)

Table S1 **Frequency of reciprocal GSVs.**
(PDF)

Text S1 **Discovery** ***P*** **-values.**
(PDF)

Text S2 ***P*** **-value inflation due to inaccurate GSV calls.**
(PDF)
