# Supplementary material for: Rare Genomic Structural Variants in Complex Disease: Lessons from the Replication of Associations with Obesity
Source: PLoS One. 2013 Mar 12;8(3):e58048. doi: 10.1371/journal.pone.0058048 (PMC3595275; doi:10.1371/journal.pone.0058048)

Supplementary Figure S1. Linkage disequilibrium in the chromosome 16p11.2 region. SNP linkage disequilibrium data (CEU) for chromosome 16p11.2 was downloaded from HapMap release 3 version R2 and displayed using Haploview version 4.2 using the standard colour scheme. Solid bars show the locations of the obesity-associated 220kb and 593kb deletions. There is no identifiable linkage disequiilibrium between the two regions.

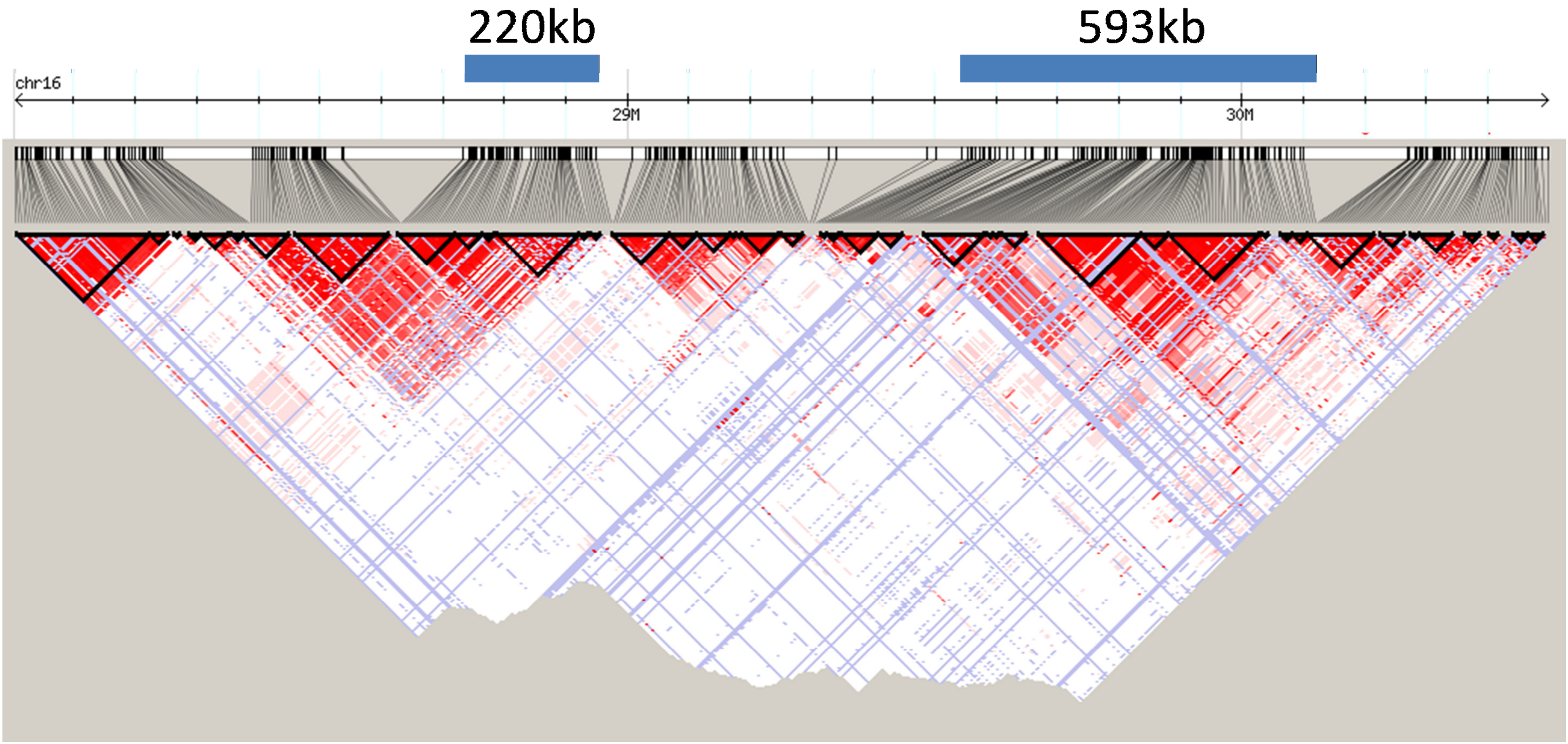

Supplement: Figure S1 — Linkage disequilibrium in the chromosome 16p11.2 region. (PDF) [file pone.0058048.s001.pdf]
