# Supplementary material for: Rare Genomic Structural Variants in Complex Disease: Lessons from the Replication of Associations with Obesity
Source: PLoS One. 2013 Mar 12;8(3):e58048. doi: 10.1371/journal.pone.0058048 (PMC3595275; doi:10.1371/journal.pone.0058048)

Supplementary Figure S2. Fasting plasma insulin in obese children stratified by age and gender. The relationships between fasting plasma insulin and BMI are shown for obese children carrying a 220kb deletion of chromosome 16p11.2 (closed circles), together with age- and gender-matched controls (open circles) from the same cohort. (a) 9 year old boys; (b) 12 year old girls; (c) 13 year old girls; (d) 14 year old boys; (e) 14 year old girls. We find no evidence for disproportionate hyperinsulinemia in deletion carriers, compared to similarly obese age- and gender-matched controls.

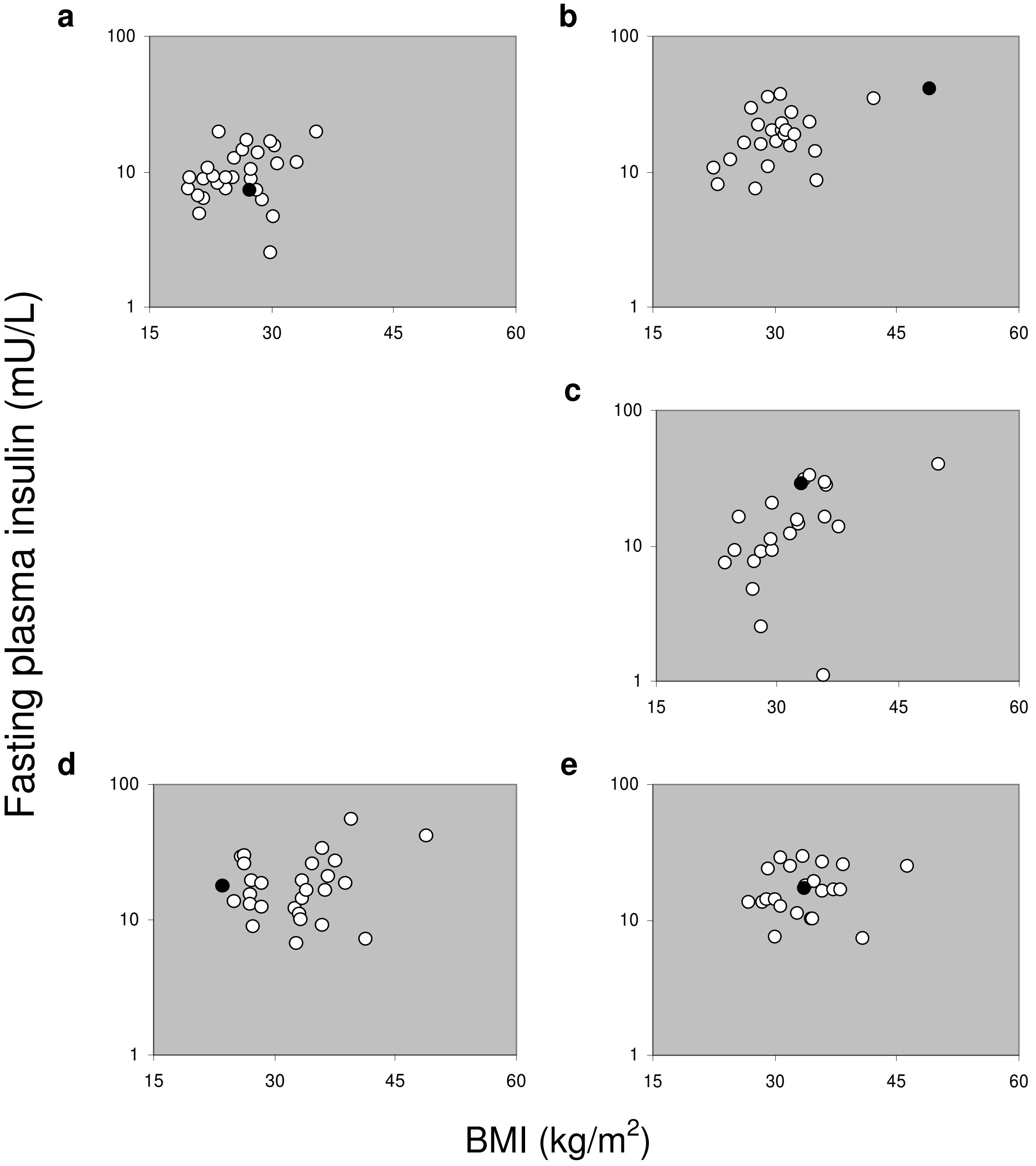

Supplement: Figure S2 — Fasting plasma insulin in obese children stratified by age and gender. (PDF) [file pone.0058048.s002.pdf]
