# Supplementary material for: Rare Genomic Structural Variants in Complex Disease: Lessons from the Replication of Associations with Obesity
Source: PLoS One. 2013 Mar 12;8(3):e58048. doi: 10.1371/journal.pone.0058048 (PMC3595275; doi:10.1371/journal.pone.0058048)

Supplementary Figure S3. SNP associations in the *FOXP2* region. Genome-wide association results in the *FOXP2* region, for BMI[11], waist-hip ratio (adjusted for BMI)[12] and insulin resistance[23], were plotted using LocusZoom (http://csg.sph.umich.edu/locuszoom). Linkage disequilibrium with relation to the top SNP (purple diamond) is according to 1000 Genomes (CEU, June 2010 release). The shading in the bottom panel corresponds to the region shown in Figure 3.

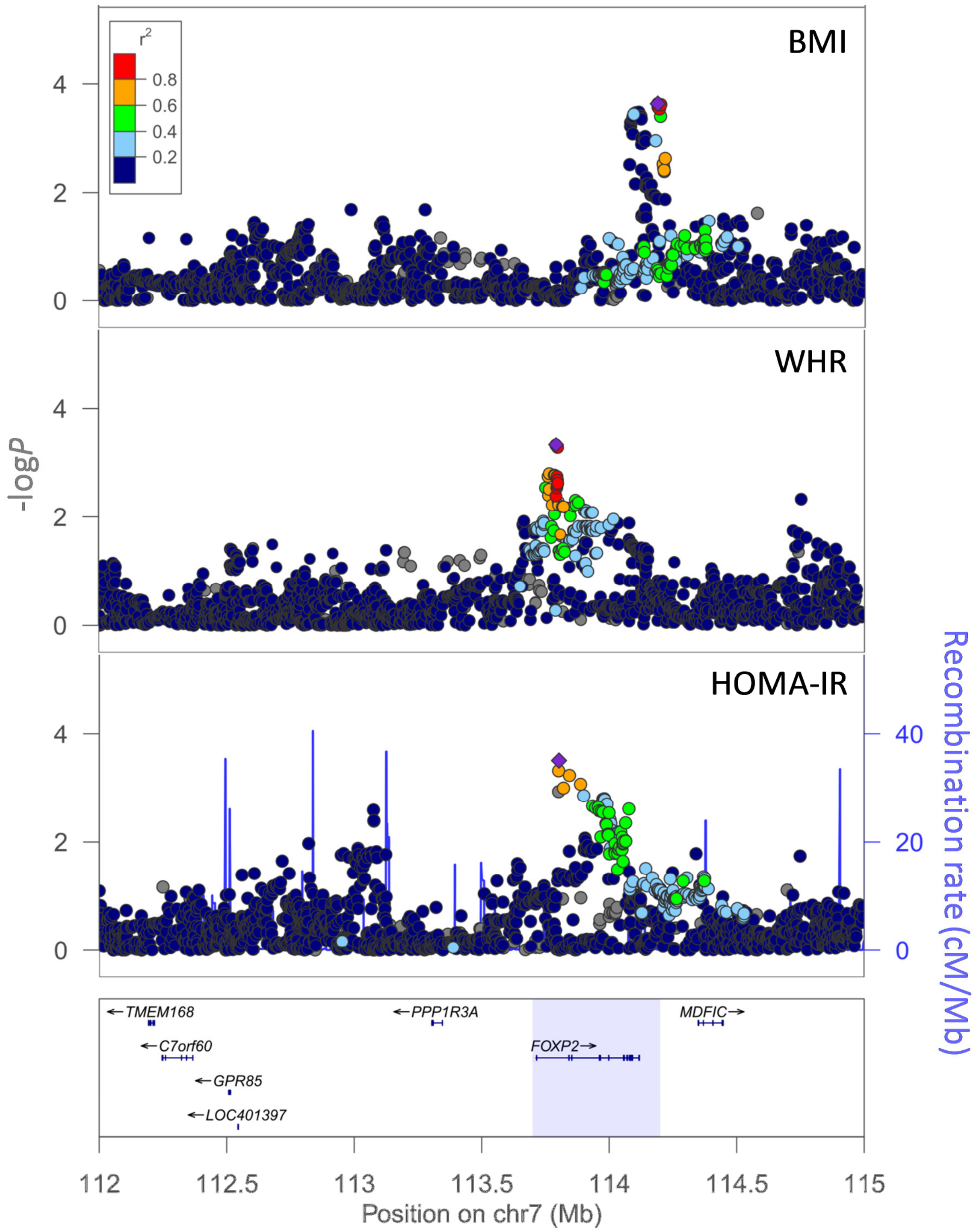

Supplement: Figure S3 — SNP associations in the FOXP2 region. (PDF) [file pone.0058048.s003.pdf]
