# Supplementary material for: Rare Genomic Structural Variants in Complex Disease: Lessons from the Replication of Associations with Obesity
Source: PLoS One. 2013 Mar 12;8(3):e58048. doi: 10.1371/journal.pone.0058048 (PMC3595275; doi:10.1371/journal.pone.0058048)
Supplement: Table S1 — Frequency of reciprocal GSVs. (PDF) [file pone.0058048.s005.pdf]

Supplementary Table S1. Frequency of reciprocal GSVs. Each cohort was analysed for the presence of GSVs reciprocal to those previously reported (i.e. deletion instead of duplication and *vice versa*), using the same methodology as for the data shown in Table 1.

	child obesity case-control		adult obesity case-control		Population cohort (NFBC1966)		
	non-obese	obese	non-obese	obese	normal	over-weight	obese
Total samples	557	645	843	701	3126	1617	470
Identified in subjects with extreme obesity							
chr3:89,250,592–89,319,536	0	0	1	0	5	3	2
chr6:52,875,284–52,892,054	0	0	0	0	0	1	0
chr8:143,268,033–143,634,461	0	0	0	0	2	0	0
chr10:541,873–818,440	0	0	0	0	0	0	0
chr11:72,013,333–72,089,312	0	0	2	0	0	0	0
chr11:105,716,030–106,419,349	0	0	0	0	0	0	0
chr15:28,700,879–30,231,488	0	0	0	0	0	0	0
chr16:28,731,428–28,951,376	1	0	1	0	1	0	2
chr17:2,224,814–2,256,880	0	0	1	0	1	1	1
chr22:49,246,176–49,313,898	0	0	0	1	0	2	1
Identified in subjects with common obesity							
chr3:104,059,109–104,092,618	0	0	0	1	4	0	1
chr5:53,467,427–53,480,255	0	0	0	0	1	0	0
chr5:77,039,051–77,076,628	0	0	0	0	0	0	0
chr5:83,835,179–83,874,339	0	0	0	0	2	1	1
chr7:20,708,193–20,711,088	4	3	0	0	4	3	1
chr7:113,843,696–113,859,679	1	0	4	0	0	0	0
chr17:49,444,406–49,449,022	0	0	1	0	3	1	0
chr19:10,489,548–10,512,171	0	0	0	0	0	0	0
