# Supplementary material for: Rare Genomic Structural Variants in Complex Disease: Lessons from the Replication of Associations with Obesity
Source: PLoS One. 2013 Mar 12;8(3):e58048. doi: 10.1371/journal.pone.0058048 (PMC3595275; doi:10.1371/journal.pone.0058048)

Supplementary Figure S4. Impact of GSV call inaccuracy on type I error rate. For a GSV with underlying population carrier frequency 0.2%, the frequency distributions for the number of GSV calls in cohorts of 1000 were determined according to the Poisson distribution ($\lambda=2$) or the Negative Binomial distribution ($r=4$, $k=\lambda/(\lambda+r)$). The marked areas indicate the GSV call frequencies in the 2 cohorts that result in type I errors for a 2-tailed test at $\alpha=0.05$, determined according to Fisher's exact test.

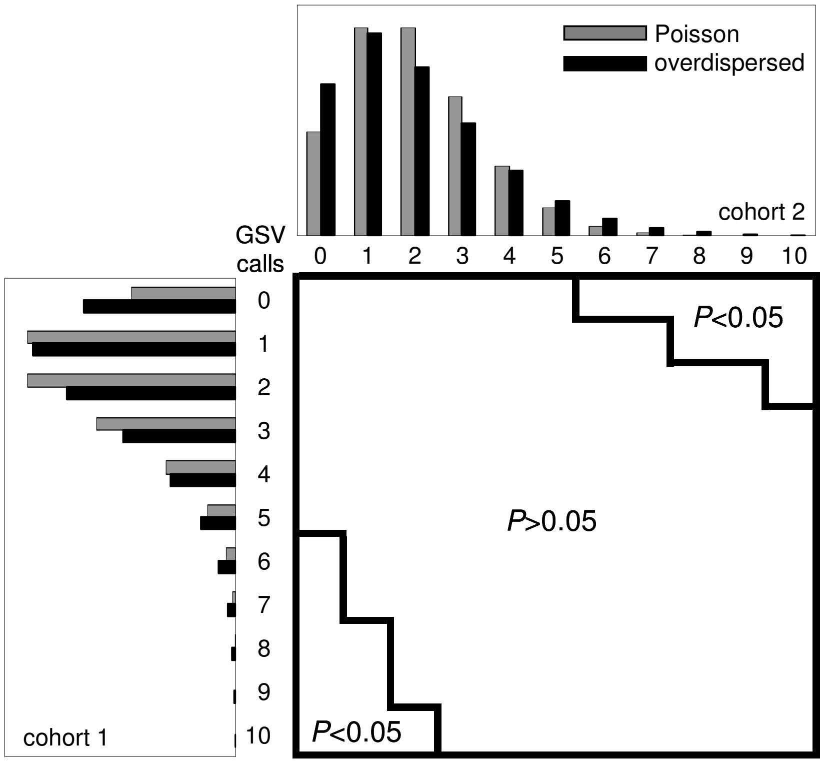

Supplement: Figure S4 — Impact of GSV call inaccuracy on type I error rate. (PDF) [file pone.0058048.s004.pdf]
