# Supplementary material for: Rare Genomic Structural Variants in Complex Disease: Lessons from the Replication of Associations with Obesity
Source: PLoS One. 2013 Mar 12;8(3):e58048. doi: 10.1371/journal.pone.0058048 (PMC3595275; doi:10.1371/journal.pone.0058048)
Supplement: Text S2 — P -value inflation due to inaccurate GSV calls. (PDF) [file pone.0058048.s007.pdf]

Supplementary Text S2

P-*value inflation due to inaccurate GSV calls*

For a rare GSV with 100% accurate detection, the frequency distribution for the number of GSV calls in a cohort is approximately Poisson. However, if GSV calls are subject to error, with both false positive and false negative calls, the frequency distribution becomes overdispersed with respect to Poisson; such a distribution, with identical mean but increased variance, can be modelled as a negative binomial distribution (also known as the gamma–Poisson (mixture) distribution): as the accuracy of GSV calling improves, the negative binomial distribution converges to the Poisson distribution such that $\mathrm{Poisson}(\lambda) = \lim_{r \to \infty} \mathrm{NB}\left(r, \frac{\lambda}{\lambda + r}\right)$, where λ is the mean number of GSV calls.

Supplementary Figure S4 illustrates the impact of inaccurate GSV calling on type I error rate, for the example of case and control of 1000 individuals each and a population frequency of GSV carriers of 0.2%. The distributions of GSV call frequencies in those cohorts are represented in 2-D, so that the occurrence of a type I error can be visualised as being located at 2 corners of this case-control space. The introduction of GSV calling error alters the distribution of GSV calls so that those regions are populated more frequently – i.e. the type I error rate increases. In the example shown, GSVs occurring according to the Poisson distribution give an empirical type I error rate at α=0.05 (according to Fisher's exact test) of 0.0051. The illustrated change in GSV calls due to calling error (due to a 50% increase in the variance of GSV calls) results in a type I error of 0.0222, a 4.4-fold increase. For lower α, the effect is even larger, e.g. 13-fold for α=0.01, 26-fold for α=0.005. Similarly, using Barnard's exact test (which for the given example is better powered, giving an empirical type I error rate of 0.041), inflation at α=0.05, 0.01 and 0.005 is, respectively, 2.0, 7.0 and 13.0.

Thus, inaccuracy in GSV calls at the level of the individual, leading to increased variance for the frequency of GSV calls, leads to inflated *P*-values and an increased rate of false-positive associations. We suggest that this is likely to apply mainly to smaller GSVs, where positive miscalls are more likely. It seems less likely to be relevant for large GSVs spanning many probes, for which false negatives calls are likely to predominate – in this case, a reduced call rate is equivalent to a reduced apparent allele frequency, resulting in reduced power and an increased type II error rate.
