# Supplementary material for: Rare Genomic Structural Variants in Complex Disease: Lessons from the Replication of Associations with Obesity
Source: PLoS One. 2013 Mar 12;8(3):e58048. doi: 10.1371/journal.pone.0058048 (PMC3595275; doi:10.1371/journal.pone.0058048)
Supplement: Text S1 — Discovery P -values. (PDF) [file pone.0058048.s006.pdf]

Supplementary Text S1

Discovery P-*values*

The original report of association with obesity of 220kb deletions of the chr16:28.73-28.95Mb region preceded our publications demonstrating that a nearby 593kb region was itself strongly associated with morbid obesity, independent of the 220kb locus. Two of 5 cases used by Bochukova, *et al.* to calculate the discovery-stage *P*-value carried extended deletions spanning both regions, and a further 4 cases and 4 controls carried the 593kb deletion alone. Due to carrying a known obesity-causing variant, therefore, these individuals were uninformative concerning a putative obesity association for the smaller region. In the light of this information, not available at the time of the original report, we have recalculated the *P*-value at discovery. Excluding the 10 uninformative subjects yields initial significance of P=4.51x10^{-4} for the 220kb deletion (3 carriers out of 278 cases; 2/7362 controls), a value that no longer survives correction for multiple testing – over 300 rare variants were identified in the discovery cohorts. Combining the discovery data with those reported for the replication obesity cohort (2/1062 cases) gives an overall *P*-value of 1.37x10^{-3} (Fisher's exact test), as shown in Table 1.

P-values at discovery for GSVs identified in subjects with common obesity by Glessner, *et al.*, as shown in Table 1, were determined using Fisher's exact test applied to the aggregate of the data from the two cohorts used in this study.
